# Optimization of fertilizer doses for increased fruit yield of dragon fruit adopting response surface methodology and Box-Behnken design

**DOI:** 10.3389/fpls.2025.1661974

**Published:** 2025-09-17

**Authors:** Ganesan Karunakaran, Sundram Ramachandran, Rangarajan Venugopalan, Rajendiran Selladurai, Manivannan Arivalagan, Anoop Kumar Srivastava, Kanakanahalli Gangadharappa Shilpa, Thimmarayappa Ruchitha

**Affiliations:** ^1^ Division of Fruit Crops, ICAR-Indian Institute of Horticultural Research, Bengaluru, Karnataka, India; ^2^ Division of Natural Resource Management, ICAR-Indian Institute of Horticultural Research, Bengaluru, Karnataka, India; ^3^ Division of Social Science and Training, ICAR-Indian Institute of Horticultural Research, Bengaluru, Karnataka, India; ^4^ ICAR-IIHR-Central Horticultural Experimental Station, Chettalli, India; ^5^ Division of Basic Science, ICAR-Indian Institute of Horticultural Research, Bengaluru, Karnataka, India; ^6^ ICAR-Central Citrus Research Institute, Nagpur, Maharashtra, India; ^7^ ICAR-IIHR, Experimental Farm, Tumakuru, Karnataka, India; ^8^ Division of Fruit Crops, ICAR-Indian Institute of Horticultural Reserach, Hesaraghatta Lake Post, Bengaluru, Karnataka, India

**Keywords:** dragon fruit, optimization of fertilizer, plant growth, nutrient acquisition, fruit yield

## Abstract

Dragon fruit stands out all among fruits not only for its essential nutrients and antioxidant content but also for its aesthetic appeal. Its high demand in consumer preferences and marketing values makes it a sought-after fruit. However, its production is limited by poor agronomic practices, particularly suboptimal use of fertilizers. Therefore, this study aims to enhance dragon fruit production by improving fertilizer use efficiency, thereby increasing fruit yield without wastage of inorganic fertilizer. To achieve this objective, two dragon fruit pulp types, white and purple-red, were chosen as test crops, and a total of three independent variables with different graded levels of N:P:K combinations were utilized. Second-order rotatable design (SORD) particularly Box-Behnken design (BBD) was employed using response surface methodology (RSM) to fix the fertilizer treatment combinations, to study the yield response and optimization of fertilizer doses. The response outcomes of white pulp fruit yield per hectare (Y_1_) and purple red pulp fruit yield per hectare (Y_2_) of dragon fruit were predicted and verified for model adequacy. The results indicated that optimum fertilizer doses (N:P_2_O_5_:K_2_O) of 400:300:650 g/pillar/year and 700:400:350 g/pillar/year for white and purple red pulped dragon fruit, respectively, led to a desirable effect, resulting in maximum yields of 25.5t ha^-1^ and 35.6t ha^-1^ for white and purple red pulped dragon fruit, respectively.

## 1 Introduction

Dragon fruit (*Hylocereus* spp.), also branded as pitahaya or pitaya, has become more and more popular in recent times due to its striking appearance and nutritional value. Originally native to Central and South America, this fascinating fruit is now grown worldwide majorly in tropical and subtropical conditions, and with a rising interest among researchers in Asia (Hoa et al., 2006; Harivaindaran et al., 2008; Suguna et al., 2011). Cultivation of dragon fruit has seen a steady rise in demand, both locally and internationally. In India, for instance, dragon fruit cultivation has expanded to over 3085 hectares in 2020 and is projected to reach 50,000 hectares in the next five years (Karunakaran et al., 2019a; Wakchaure et al., 2021). Its cultivation is widespread across states such as Karnataka, Gujarat, Maharashtra, Andhra Pradesh, Tamil Nadu, Kerala, Andaman & Nicobar Islands,West Bengal, Odisha, and Telangana with recent expansions into Madhya Pradesh, Punjab, Haryana, Rajasthan, Uttar Pradesh and North Eastern States (Karunakaran et al., 2019b).

The cultivation of dragon fruit demands vigilant attention to soil quality, watering practices, and pest control measures. To attain heightened yields, it becomes imperative to provide the fruit with an array of essential nutrients (Grusak et al., 2016), which can be effectively supplied through soil amendments and inorganic fertilizers. Ensuring adequate soil fertility fosters robust plant growth, vigorous flowering, and abundant fruit yield. Nonetheless, the lack of comprehensive fertilizer management information within dragon fruit orchards has led to yield disparities ranging from 2–9 t ha^-1^ (Fernandes et al., 2018; Moreira et al., 2012). Furthermore, dragon fruit’s resilient nature and adaptability to diverse soil and climatic conditions necessitate balanced fertilization to meet its growth requirements and ensure satisfactory yields (Alves et al., 2021). However, the precise nutrient demands of dragon fruit remain elusive (Mizrahi, 2014). Notably, fruit yield and growth factors are considerably improved by addition of nitrogen and phosphorus fertilizers and interaction effects of N and P have increased shoot numbers, flowering, and fruit set (Hariyanto et al., 2023). Potassium, being a crucial nutrient in numerous biochemical processes, particularly influences the quality of dragon fruit (Then, 2013).

Poor soil fertility and improper fertilizer management lead to reduction in dragon fruit plant growth and development, fruit yield as well as fruit quality (IPNI, 2014; Perween, 2017; Karunakaran et al., 2021a, b; Lima et al., 2021). Gomez and Zorita (2015) have analyzed the nutrient contents in plant tissue and fruits of six different varieties of dragon fruit and reported that potassium is the nutrient most accumulated in Pitaya followed by N and P, hence inadequate and imbalance supply of these nutrients influence fruit yield and quality. Likewise, addition of potassium fertilizer boosted dragon fruits production by 20-24% and 64-70% in the first and second years, correspondingly (Fernandes et al., 2018). Omitting N, P, and K from recommended doses of fertilizers led to yield reductions in the tune of -50%, 28%, and 29%, respectively has been reported (IPNI, 2014). Moreover, cutting K fertilizer by half caused the fruit yield reduction by 9%, whereas rising K fertilizer dose by half augmented fruit yield by 2.5% when compared with optimum dose. Therefore, optimizing doses of N:P: K fertilizers are inevitable to enhance the yield of dragon fruit.

Moreover, a comprehensive understanding of fertilization techniques in crop management aids in expanding dragon fruit cultivation areas. Furthermore, nutrient requirements vary among dragon fruit species, emphasizing the importance of accurately assessing nutrient needs and understanding the species’ nutrient status in nurseries. While numerous studies have recommended various N:P:K doses through randomized combinations and statistical analyses, none have proposed an optimal N:P:K dose in India. Hence, this study aims to determine optimal N:P:K dose for white- pulped and purple-red-pulped dragon fruits to achieve higher yields, utilizing the Box-Behnken Design and Response Surface Methodology (RSM) to elucidate relationship between response and independent variables.

The RSM emerges as an extensively employed statistical tool for optimizing processes by identifying key factorial variables. It plays a pivotal role in enhancing performance of a system and in boosting efficiency of process without escalating cost and time (Sujayasree et al., 2022; Cano-Lamadrid et al., 2023). Specifically, in determining the optimal NPK nutrient dose for dragon fruit, RSM serves as a crucial tool for establishing the relationship between response and independent variables. Typically, this method is employed to delineate a response surface within a specified area of interest or under selected working conditions to meet the purpose (Box and Draper, 1965; Myers et al., 2016; Beegum et al., 2018; Pandiselvam et al., 2022). In this study, wide ranges of N (200, 450 and 700g), P (150, 275 and 400g) and K (300, 650 and 1000g) are applied to both the dragon fruit types in 13 treatment combinations adopting RSM. The objective of the current study is optimization of N:P:K doses for red-purpled and white- purpled dragon fruits under nominal soil environment conditions.

## 2 Materials and methods

The field experiment was carried out at ICAR-IIHR-Central Horticultural Experiment Station (CHES), Hirehalli, Tumkur District, Karnataka, India, from 2020–21 to 2022-23. The GPS coordinates of station is 13° 16’ 27.22” N and 77° 11’ 1.32” E, with an altitude of 845m form mean sea level. The annual rainfall of the station is 800mm, and the mean maximum and minimum air temperatures are 30.3°C and 20.7°C, respectively. The initial soil properties of experimental soil are portrayed in **Table 1**. The soil has clay loam texture and medium organic matter content with slightly acidic reaction, deficient in nitrogen and sulphur; and sufficient with respect to all other nutrients.

**Table 1:** Initial physico-chemical properties of experimental soil.

Parameters	Value	Remarks	Reference
Texture	CL	–	USDA, 1987
pH (1:2.5)	6.51	Slightly acidic	Jackson, 1973
Electrical conductivity (dS m^-1^)	0.38	Normal	
Organic carbon content (g/kg)	7.20	Medium	Walkley and Black, 1934
Available nitrogen (kg ha^-1^)	267	Low	Subbiah and Asija, 1956
Available phosphorus (kg ha^-1^)	21.7	Medium	Bray and Kurtz, 1945
Available potassium (kg ha^-1^)	458	High	Hanway and Heidel, 1952
Available calcium [cmol(p^+^) kg^-1^]	7.15	Sufficient	Jackson, 1958
Available magnesium [cmol(p^+^) kg^-1^]	2.10	Sufficient	
Available sulphur (kg ha^-1^)	17.9	Low	Chesnin and Yien, 1951
Available iron (mg kg^-1^)	13.6	Sufficient	Lindsay and Norvell, 1978
Available manganese (mg kg^-1^)	14.0	Sufficient	
Available copper (mg kg^-1^)	1.77	Sufficient	
Available zinc (mg kg^-1^)	0.85	Sufficient	

*CL- Clay loam.

In March, 2017; four plants were planted all the four sides of a pillar, and the pillars were erected maintaining 3.0m inter row distance and 2.5m intra row distance. Over the first three years period (2017-2020), the plants were allowed to grow and progress to the fruiting stage. Subsequently, from the fourth year onwards, from 2020–21 to 2022-23, during the fruit harvesting season spanning from May to October, the fruits were harvested, and the yield of each pillar was meticulously recorded.

**Figure 1:**
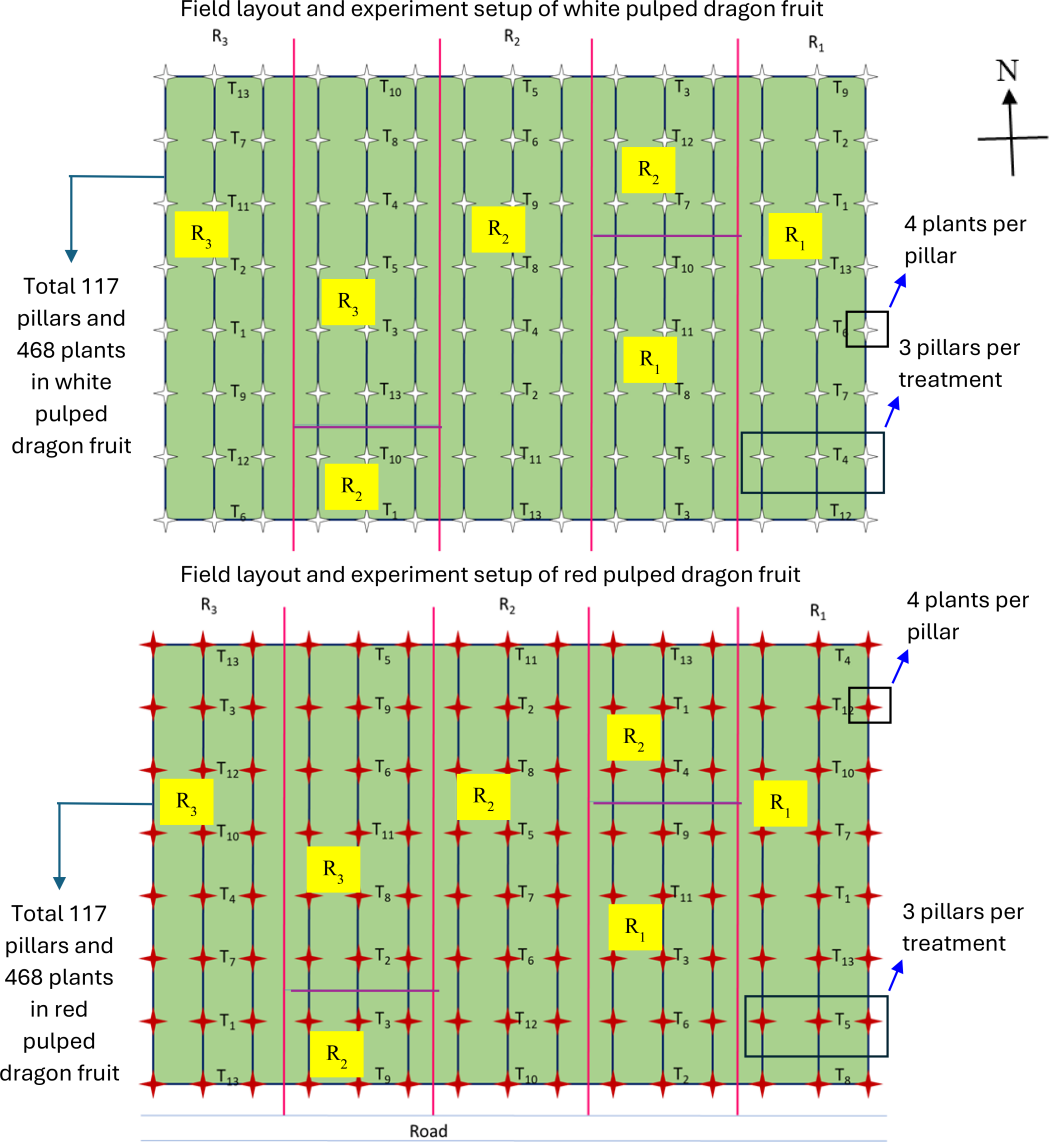
Field layout and experiment setup of dragon fruit nutrient management trial (15 rows; treatments 13 (T_1_-T_13_); 3 replications/blocks (R_1_-R_3_); 3 pillars per treatment, and 4 plants per pillar; in total 117 pillars and 468 plants for each dragon fruit type; red color star indicates red-pulped dragon fruit pillars and white color star indicates white pulped dragon fruit pillars; treatments are randomly allotted).

The experiment was conducted in white and purple-red pulped dragon fruit orchards (**Figure 1**), employing various levels of N:P:K doses using a second-order rotatable design (SORD) with thirteen treatment combinations (**Tables 2**, **3**) and three replications. The treatments (N:P_2_O_5_:K_2_O combinations) included

T_1_ = 200:400:650 g/pillar,
T_2_ = 200:400:650 g/pillar,
T_3_ = 450:400:1000 g/pillar,
T_4_ = 700:275:1000 g/pillar,
T_5_ = 700:400:650 g/pillar,
T_6_ = 450:400:300 g/pillar,
T_7_ = 200:275:300 g/pillar,
T_8_ = 200:275:1000 g/pillar,
T_9_ = 700:275:300 g/pillar,
T_10_ = 450:150:300 g/pillar,
T_11_ = 450:150:1000 g/pillar,
T_12_ = 700:150:650 g/pillar, and
T_13_ = 450:275:650 g/pillar.

**Table 2:** Coded and encoded values of independent variables and their levels adopted in the experiment.

Independent variables	Coded levels	-1	0	+1
Nitrogen (N)	X_1_	200	450	750
Phosphorus (P_2_O_5)_	X_2_	150	275	400
Potassium (K_2_O)	X_3_	300	650	1000

**Table 3:** The experiment design matrix (treatments with independent variables) and pooled yield data of dragon fruit (observed values of response variable).

Treatments	Independent variables			Yield-White pulped (t ha^-1^)				Yield-Red pulped (t ha^-1^)			
	N (g/pillar), X_1_	P_2_O_5_ (g/pillar), X_2_	K_2_O (g/pillar), X_3_	2020	2021	2022	Pooled (Y_1_)	2020	2021	2022	Pooled (Y_2_)
T_1_	200	400	650	19.5	27.3	24.6	23.8	19.2	41.1	36.8	32.3
T_2_	200	150	650	22.0	28.3	25.4	25.3	21.0	43.3	42.6	35.6
T_3_	450	400	1000	17.1	17.2	17.5	17.2	20.1	38.5	41.3	33.3
T_4_	700	275	1000	19.7	25.3	29.8	24.9	20.0	36.9	36.2	31.0
T_5_	700	400	650	15.8	16.4	18.1	16.8	21.4	44.4	39.8	35.2
T_6_	450	400	300	19.9	29.3	22.0	23.7	21.3	35.0	39.4	31.9
T_7_	200	275	300	19.3	25.7	25.9	23.6	19.0	33.6	38.6	30.4
T_8_	200	275	1000	15.3	18.9	26.7	20.3	19.6	39.6	40.9	33.4
T_9_	700	275	300	16.8	26.1	20.4	21.1	20.5	43.6	44.2	36.1
T_10_	450	150	300	16.3	22.1	27.0	21.8	20.6	36.2	39.1	31.9
T_11_	450	150	1000	16.4	21.6	21.6	19.9	19.8	40.8	34.2	31.6
T_12_	700	150	650	22.0	27.4	27.0	25.5	22.4	35.4	41.2	33.0
T_13_	450	275	650	19.9	25.7	29.8	25.1	18.7	36.8	46.2	33.9
SE	–	–	–	–	–	–	1.86	–	3.41	–	–
CD @ 5%	–	–	–	NS	NS	NS	3.83	NS	7.04	NS	NS

X_1_, X_2_, X_3_: Coded independent variables; Y_1_: Fruit yield (t ha^-1^) (white pulp); Y_2_: Fruit yield (t ha^-1^) (purple-red pulp).

In each treatment, respective nutrient doses were administered in four splits (S) with the following proportions: S_1_ in March (10:40:40), S_2_ in June (20:10:45), S_3_ in October (40:30:0), and S_4_ in January (30:20:15). For instances, 10:40:40 means 10% of total N; 40% of total P_2_O_5_ and 40% of total K_2_O in particular treatment, similarly for other proportions were followed in all the treatments. All the N, P and K nutrients were supplied through urea (contains 46% N), single super phosphate (contains 16% P_2_O_5_), and muriate of potash (contains 60% K_2_O) fertilizers, respectively. Since sulphur was deficient in the soil, sulphur requirement of production system was met through supplying single super phosphate (that contains 11% S).

Furthermore, alongside fertilizer application, each treatment received a uniform supply of farmyard manure (10kg), and neem cake (1kg) per pillar, divided equally between March and October of each year. Irrigation was administered via drip irrigation at a rate of 4 liters per pillar, twice a week. All selected plants exhibited nearly uniform growth and vigor and received consistent cultural practices. Observations on the total fruit yield per pillar per year were documented throughout the experimental duration and converted to tons per hectare per year (**Table 3**). The yield data were documented for the years 2020/21, 2021/22, and 2022/23 for experimental purposes, and these three-year data were amalgamated for the entire season of each harvest year, forming the basis for analysis in this experiment (**Table 3**).

The response surface methodology (RSM) is a statistical method to study the response and Box-Behnken design or second-order polynomial model is mathematical/statistical tool used to predict the yield and optimize the fertilizer doses based on the response obtained in the RSM. Statistical analysis was conducted using SAS V 9.3 (2012) for calculation of analysis of variance (ANOVA) (Montgomery, 2017). The same software had been used for Box-Behnken design to establish a second-order polynomial model using yield responses under various N:P:K combinations (Montgomery, 2017). The proposed second-order polynomial model for predicting response variable is depicted as below (Equation 1):


(1) $$\text{Y}={\text{B}}_{0}+{\text{B}}_{1}{\text{X}}_{1}+{\text{B}}_{2}{\text{X}}_{2}+{\text{B}}_{3}{\text{X}}_{3}+{\text{B}}_{12}{\text{X}}_{1}{\text{X}}_{2}+{\text{B}}_{13}{\text{X}}_{1}{\text{X}}_{3}+{\text{B}}_{23}{\text{X}}_{2}{\text{X}}_{3}+{\text{B}}_{11}{{\text{X}}_{1}}^{2}+{\text{B}}_{22}{{\text{X}}_{2}}^{2}+{\text{B}}_{33}{{\text{X}}_{3}}^{2}$$

Here, Y corresponds to the dependent or response variable, and Xi (i=1-3) stands for the independent variables (X_1_=N, X_2_=P_2_O_5_, X_3_=K_2_O, **Table 3**) (Myers et al., 2016; Montgomery, 2017). Coefficients of this model are designated by B_0_ (constant), B_1_, B_2_ and B_3_ (linear coefficients), B_11_, B_22_ and B_32_ (quadratic coefficients), and B_12_, B_13_ and B_23_ (interactive coefficients) (**Table 4**). The actual second-order polynomial models obtained through this study for predicting the dragon fruit yield with different coefficients are depicted in Equation 2 and Equation 3. The effects of interaction of the independent variables are portrayed in 3-D pictorial form. The fitness of the model was further confirmed using coefficient of determination (R^2^), lack of fit and F test values.

**Table 4:** Regression coefficients of adopted model for optimizing N:P:K doses for higher yield.

Coefficients	Y_1_	Y_2_
Intercept (B_0_)	25.10 ^S^	33.95^S^
B_1_	-0.746 ^NS^	0.569 ^NS^
B_2_	-1.54 ^NS^	0.213 ^NS^
B_3_	-0.98^NS^	-0.328 ^NS^
B_12_	-1.464 ^NS^	1.513 ^NS^
B_13_	1.775 ^NS^	-2.228^S^
B_23_	-1.150 ^NS^	0.425 ^NS^
B_11_	-0.054 ^NS^	0.408 ^NS^
B_22_	-1.879 ^NS^	-0.213 ^NS^
B_33_	-2.571 ^NS^	-1.513 ^NS^

*Significant at 5% by t-test: S-Significant; NS – Non-significant.

### 2.1 Prediction model for white pulp fruit yield (Y_1_)


(2) $$Y_{1}:25.10+0.746*[({\text{X}}_{1}–450)/250]+-1.543*[({\text{X}}_{2}–275)/125)]+-0.988*[({\text{X}}_{3}-650)/350)]+[({\text{X}}_{1}–450)/250)]*[({\text{X}}_{2}–275)/125)]*-1.464+[({\text{X}}_{1}–450)/250)]*[({\text{X}}_{3}–650)/350)]*1.775+[({\text{X}}_{2}–275)/125)]*[({\text{X}}_{3}–650)/350)]*-1.150+[({\text{X}}_{1}–450)/250)]*[({\text{X}}_{1}–450)/250)]*-0.054+[({\text{X}}_{2}–275)/125)]*[({\text{X}}_{2}–275)/125)]*-1.879+[({\text{X}}_{3}–650)/350)]*[({\text{X}}_{3}–650)/350)]*-2.571$$

### 2.2 Prediction model for purple-red pulp fruit yield (Y_2_)


(3) $$Y_{2}:33.95+0.569*[({\text{X}}_{1}–475)/275)]+0.213*[({\text{X}}_{2}–275)/125)]+-0.328*[({\text{X}}_{3}-650)/350)]+[({\text{X}}_{1}–475)/275)]*[({\text{X}}_{2}–275)/125)]*1.513+[({\text{X}}_{1}–475)/275)]*[({\text{X}}_{3}–650)/350)]*-2.228+[({\text{X}}_{2}–275)/125)]*[({\text{X}}_{3}–650)/350)]*-0.425+[({\text{X}}_{1}–475)/275)]*[({\text{X}}_{1}–475)/275)]*0.408+[({\text{X}}_{2}–275)/125)]*[({\text{X}}_{2}–275)/125)]*-0.213+[({\text{X}}_{3}–650)/350)]*[({\text{X}}_{3}–650)/350)]*-1.513$$

Where, Y_1_: White pulp dragon fruit yield per ha; Y_2_: Purple red pulp dragon fruit yield per ha; X_1_: Nitrogen (g/pillar); X_2_: Phosphorus (g/pillar); X_3_: Potassium (g/pillar).

## 3 Results and discussion

The year-wise and pooled fruit yield data are summarized in **Table 3**. The pooled mean data of fruit yield of white-pulped dragon fruit to different treatments/fertilizer combinations showed a significant effect at 5% significance level, while the purple-red-pulped variety did not show significant results. These findings may not fully reflect the optimal nutrient requirements for maximizing yield. Therefore, a predictive model was employed to determine the precise nutrient dosage needed for dragon fruit cultivation. The data of response variables and different nutrient combinations as input variables were used to develop a generalized second-order polynomial model for predicting the yield response. This model employed multiple regression techniques to analyze the data and create a response surface model. The adequacy of the models, with or without interactions of the dependent variables, was evaluated using R² (coefficient of determination). Additionally, the obtained model was evaluated with the help of modified coefficient of determination (R² modified) and lack of fit probability (p). The adequacy of the model for fruit yield was tested using ANOVA. The non-significant F values of lack of fit (<0.05) had confirmed strength of the model. Competency was further assessed using ANOVA as detailed in **Table 5**.

**Table 5:** Statistical parameters and ANOVA of the second order polynomial model for yield response in dragon fruits. .

Statistical parameters	Y_1_	Y_2_
Mean of response (t ha^-1^)	22.54	33.05
RMSE	2.60	1.06
R^2^	0.71	0.91
Model F value	2.24	3.396
P value	< 0.05^S^	< 0.05^S^
Lack of fit	>0.05^NS^	>0.05^NS^

S, Significant at 5%; NS, Non-significant.

### 3.1 Effect of fertilizer doses on fruit yield

Plant biological parameters are pivotal in determining the effects of applied nutrients, making it essential to optimize nutrient requirements for Dragon fruit. The pooled data in **Table 3** indicates that Treatment-T_12_ (700g N, 150g P_2_O_5_, and 650g K_2_O per pillar per year) achieved the highest and significant yield of 25.5t ha^-1^ for white-pulped dragon fruit. Conversely, Treatment-T_9_ (700g N, 275g P_2_O_5_, and 300g K_2_O per pillar per year) yielded the highest (36.1t ha^-1^) for purple-red-pulped dragon fruit though no significant difference was found among treatments.

In the experimental setup, white-pulped dragon fruit yields (Y_1_) varied significantly, ranging from 16.8 to 25.5t ha^-1^ across different nutrient combinations. Similarly, purple-red-pulped dragon fruit yields (Y_2_) varied from 30.4 to 36.1t ha^-1^. This showed that purple-red pulped dragon fruit largely responded to applied fertilizers than the white pulped dragon fruit in terms of plant growth and higher yield. Moreover, these yield differences might have stem from the varying growth responses and yield potentials of the two species, influenced by the nutrient combinations used. To pinpoint optimal nutrient doses, a predictive model was developed using a three-dimensional response surface methodology (RSM) to analyze various N:P:K combinations. The multiple regression analysis identified the optimal fertilizer nutrient combinations for higher yields: 400:300:650 g N:P_2_O_5_:K_2_O per pillar/year for white dragon fruits and 700:400:350 g N:P_2_O_5_:K_2_O per pillar/year for purple-red dragon fruits. These findings highlighted that while RSM can effectively predict optimal nutrient combinations based on the yield responses from all the 13 treatment or fertilizer combinations. This method could be more appropriate than the maximum yield obtained in single nutrient combination. Among these nutrient combinations, nitrogen and potassium individually contributed most significantly towards yield, while their quadratic interactions were not statistically significant (p<0.05) (**Table 4**). Notably, application of N:P:K fertilizer substantially increased fruit yield compared to conditions without N:P:K fertilization, as supported by previous research (Nasreen et al., 2013; Chakma et al., 2014).

In white-pulped dragon fruit, potassium emerged as a critical nutrient, surpassing nitrogen and phosphorus requirements. A significant association between potassium and fruit development was also reported in fruit crops (Wang et al., 2017). Likewise, Gomez and Zorita (2015) and Fernandes et al. (2018) had reported that potassium is the nutrient most accumulated in dragon fruit followed by nitrogen and phosphorus. Potassium involves in maintaining ionic balance in the cytoplasm, thereby promoting cell elongation (Hu et al., 2016). Additionally, K levels have been shown to influence both dragon fruit yield and quality (Sahu et al., 2022). In specific cases, the interaction between nitrogen and potassium was found to boost strawberry fruit yield (Rangel et al., 2020). Their study highlighted that a concentration of nitrates in fruits at 12 to 15mol m^-3^ of NO_3_^-^, coupled with 7mol m^-3^ of K^+^, yielded the most favorable interaction, leading to increased fruit yield. However, in contrast to these results, in purple-red pulped dragon fruit nitrogen is found to be critical along with phosphorus and potassium. The similar results of increased fruit yield and growth factors by addition of nitrogen and phosphorus fertilizers and interaction effects were reported by earlier workers (Hariyanto et al., 2023; IPNI, 2014). Omission of N in fertilizer schedule caused the dragon fruit yield reductions in the tune of 50%, when compared to 28% and 29% in cases of P and K, respectively (IPNI, 2014). This showed the importance of N, P, and K on dragon fruit production.

### 3.2 Second-order polynomial model for yield response

**Figure 2:**
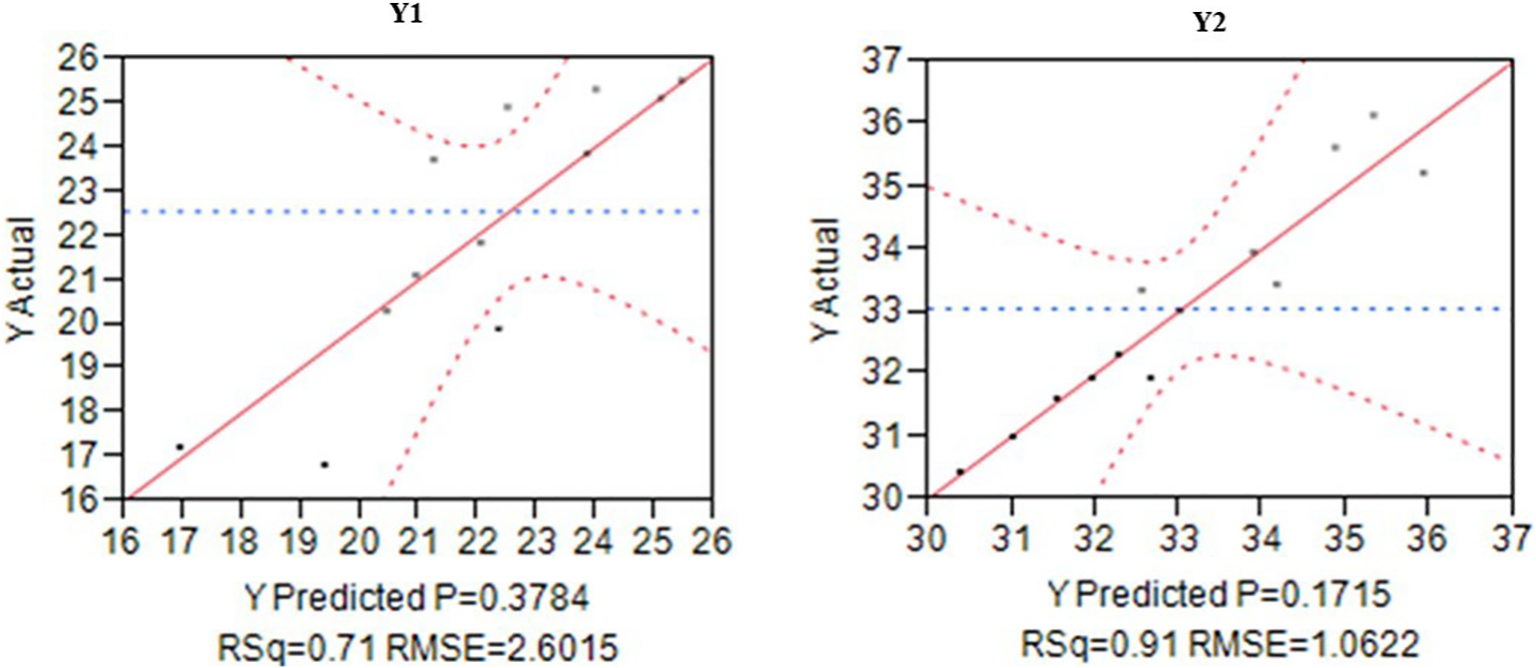
Actual vs. Predicted plot for fruit yield of white (Y1) and purple-red pulped (Y2) dragon fruits.

The second-order polynomial regression model was established using RSM with three independent variables. The prediction models are depicted as Equation 2 and Equation 3 for white and purple-red pulped dragon fruits, respectively. The model’s significance (P<0.05) was affirmed by the response from the second-order response surface model. Linear, interactive and quadratic expressions of fruit yield exhibited significance (P < 0.05) of prediction models (R^2^ = 0.71 and 0.91) (**Figure 2**). However, the interaction among nutrient factors, as indicated by the t-test values, was estimated to be not significant (P < 0.05) (**Table 4**). Independent nutrient variables such as N, P, and K in the interaction process, as depicted in the plot, validated the preference of RSM with interaction effect as created. Effect of variable response is illustrated by sign and magnitudes of the coefficient. A positive and negative sign denotes an increase and a decrease in response, respectively. When the interactions are significant, interactive variables can be increased to a level to attain the constant values of the response (Draper and Hunter, 1966; Montgomery, 2004). The levels of nutrients in both linear and interaction terms exhibit a positive as well as negative correlations (**Table 4**). Increasing the quantity of fertilizer may be attributed to the increase in fruit yield up to certain level. This finding was in consistent with other studies (Li et al., 2014; Perween and Hasan, 2019).

### 3.3 Summary statistics of fit and plot analysis of actual vs. predicted

The experiment involved combinations of fertilizer doses, with the Response Surface Methodology (RSM) capturing 71.0% and 91.0% of the white and purple red pulped dragon fruit yield, respectively, as evidenced by the graphical representation. The dotted lines closely aligning with the mean line indicate the model’s significance in 71% and 91% of Y_1_ and Y_2_, respectively (**Figure 2**). Results from the ANOVA table indicate that the model means of response values were 22.54 and 33.05, with R² values of 0.71 and 0.91, and a RMSE (Root Mean Square Error) of 2.6 and 1.06, suggesting minimal prediction error for these nutrient levels and confirming the model’s good fit for obtaining right yield response (**Table 5**). Non-significant F values for lack of fit (p<0.05) hold good for the competency of model.

### 3.4 Optimization of fertilizer doses for fruit yield using RSM

**Figure 3:**
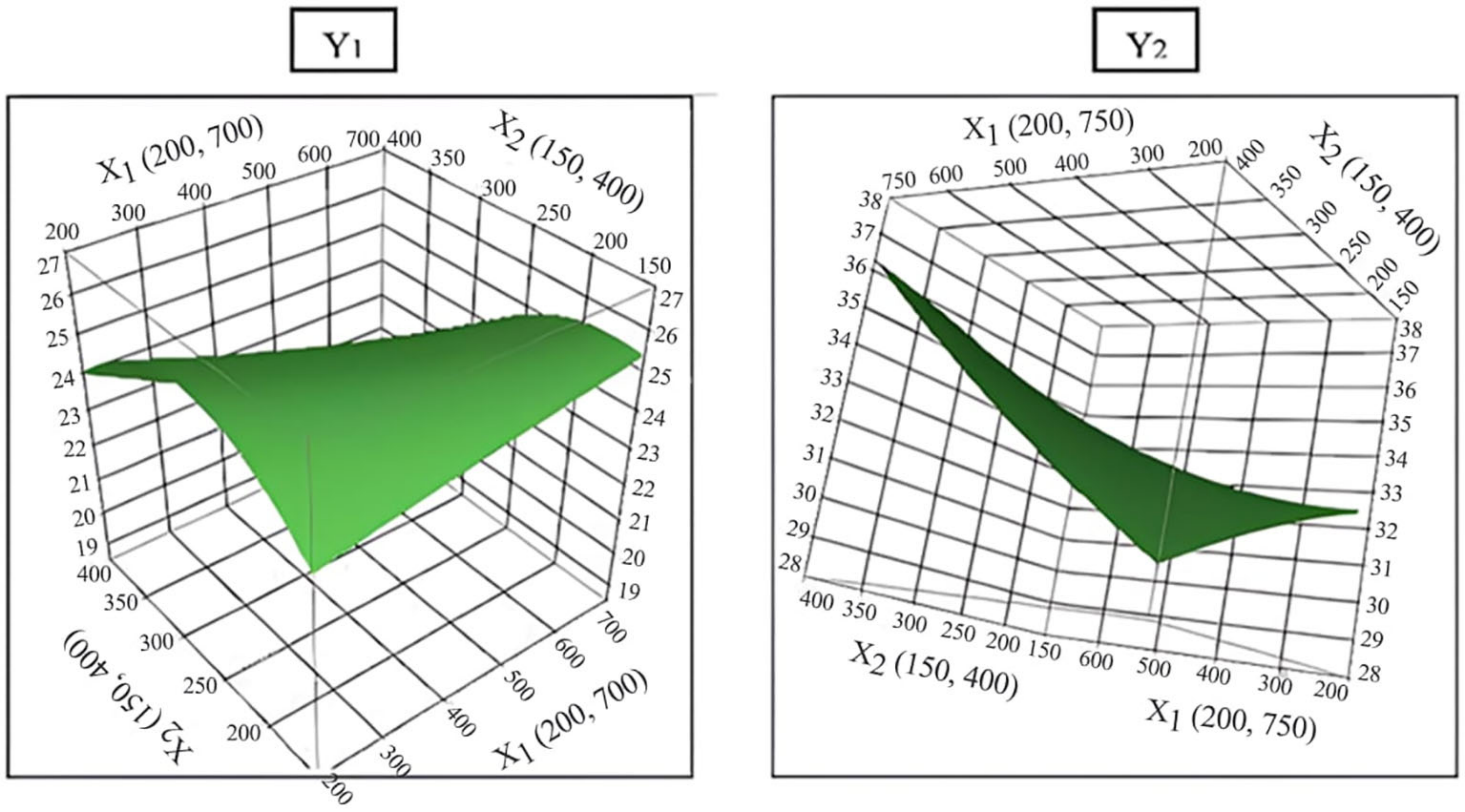
Optimization of three independent variables using the quadratic response surface plot for white pulp fruit yield (Y_1_) and purple-red pulp fruit yield (Y_2_).

**Figure 4:**
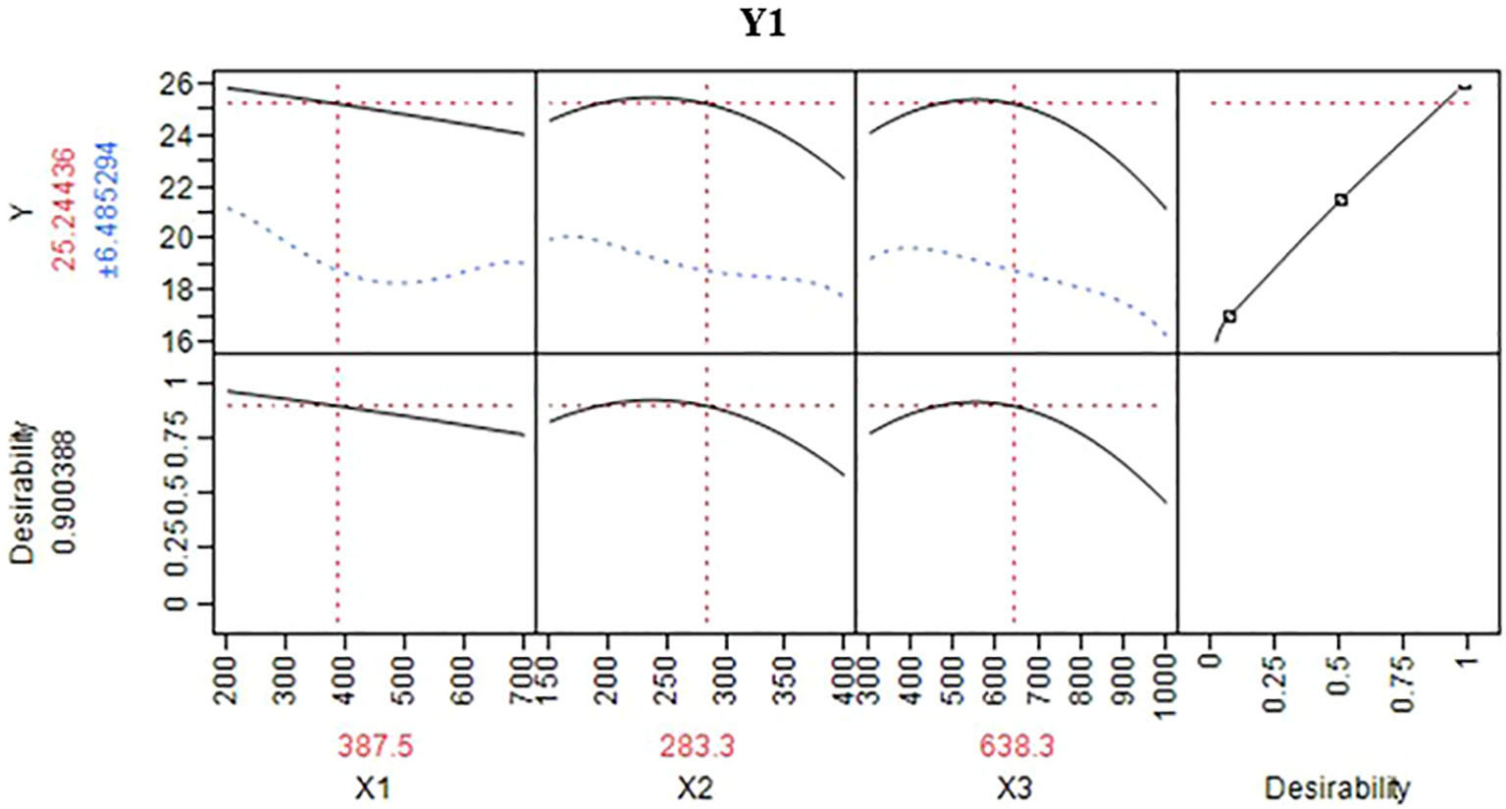
Prediction profiler for optimized conditions of fruit yield of white pulped dragon fruit per pillar (Y_1_).

**Figure 5:**
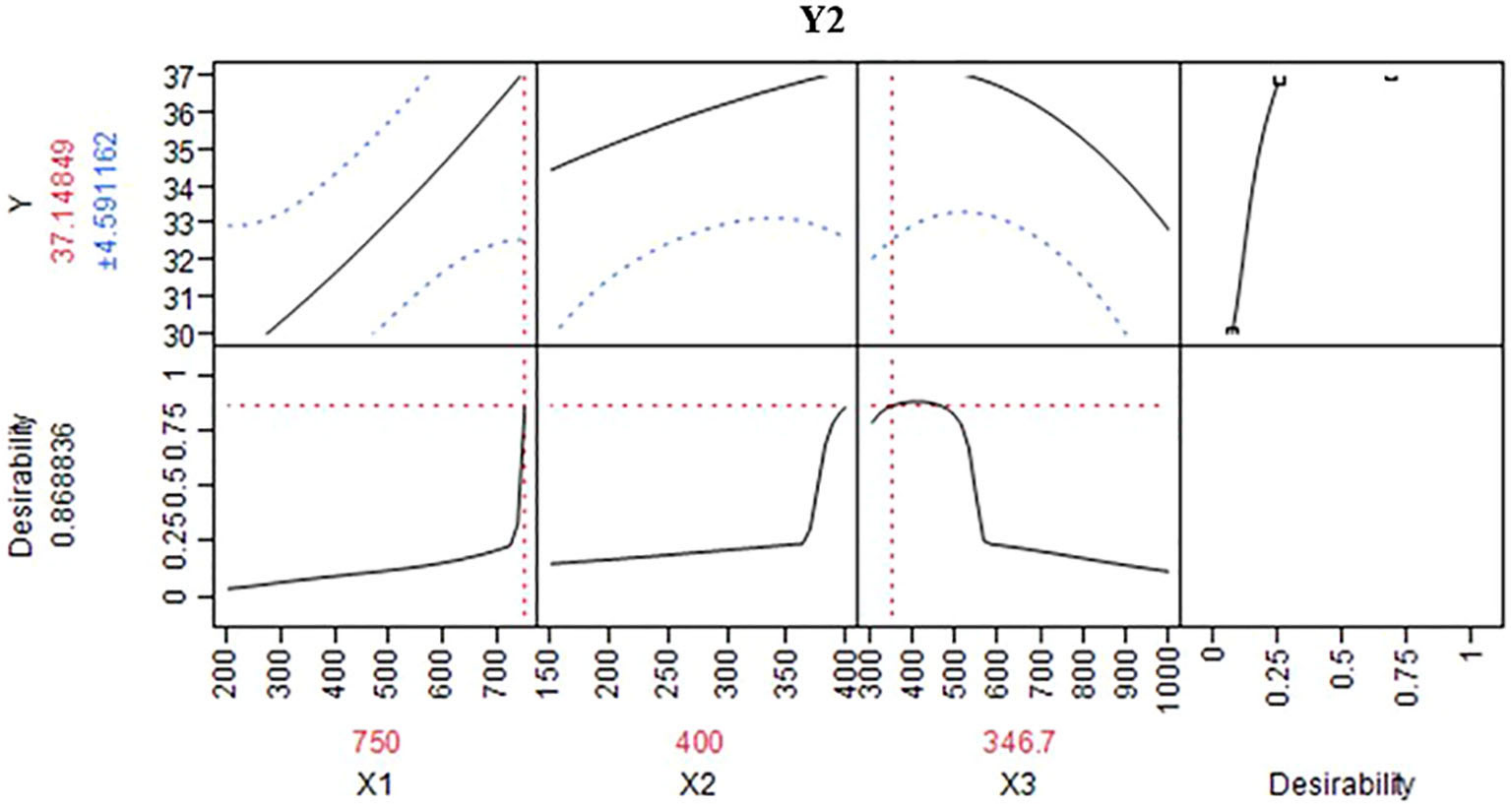
Prediction profiler for optimized conditions of fruit yield of purple-red pulped dragon fruit per pillar (Y_2_).

Intention of this study was to determine optimal N:P:K levels for maximizing the dragon fruit yield. To achieve this, a selected model was utilized (Ade Omowaye et al., 2021; Romero et al., 2022). This predictive model generated information and a three-dimensional response surface plot for various N:P:K combinations, illustrating the trend of response variation within the chosen range of input variables, as depicted in **Figure 3**. This figure shows interrelationship between response (fruit yield) and independent (N:P:K doses) variables (Bezerra et al., 2008; Yuan et al., 2018). Further through this the range of constant variables (fruit yield) can be illustrated in the plane of the independent variables adopting a two-dimensional surface plot (Alam and Singh, 2010; Shameena et al., 2019). The response surface plot (**Figure 3**) demonstrates effect of N:P:K nutrients on white pulped dragon fruit yield (Y_1_) and purple-red pulped dragon fruit yield (Y_2_). The shape of the surface helps locate the optimal range with reasonable precision. The results indicated that the optimal nutrient (N:P:K) doses for maximum yield (25.5t ha^-1^), corresponding to 388, 283, and 638g per pillar in white pulped dragon fruit, with a desirability of 90% (**Figure 4**). In case of purple red pulped dragon fruit, the optimal doses of N:P:K were 750, 400 and 347g per pillar per year to achieve higher yield of 37.1t ha^-1^, with a desirability of 87% (**Figure 5**). This study demonstrates the possibility of determining the optimum settings of independent variables for maximizing the response variable (**Table 6**). It also suggests that applying higher doses of nitrogen and potassium, rather than phosphorus, can result in a maximum fruit yield. This finding is in consistent with the previous study of increase in fruit yield with application of N:P:K (Monga et al., 2004; Maity et al., 2006; Oloyede et al., 2013). Whereas minimum fruit yield was observed with native fertility (Sarker and Rahim, 2012; Nasreen et al., 2013). Overall, the results suggest that response surface methodology could be a cost-effective alternative for optimizing input variables before conducting field experiments to validate predicted values.

**Table 6:** Predicted and experimental values of response (yield) at variable N:P:K doses.

Treatment	Observed Responses		Predicted (Y_1p_) (t ha^-1^)	Difference (Y_1_-Y_1p_) (t ha^-1^)	Predicted (Y_2p_) (t ha^-1^)	Difference (Y_2_ -Y_2p_) (t ha^-1^)
	Y_1_ (t ha^-1^)	Y_2_ (t ha^-1^)				
T_1_	23.8	32.3	23.8	0	32.3	0
T_2_	25.3	35.6	24.0	1.3	34.9	0.7
T_3_	17.2	33.3	17.0	0.2	32.6	0.7
T_4_	24.9	31.0	22.5	2.4	31.0	0
T_5_	16.8	35.2	19.4	-2.6	35.9	-0.7
T_6_	23.7	31.9	21.2	2.5	32.0	-0.1
T_7_	23.6	30.4	26.0	-2.4	30.4	0
T_8_	20.3	33.4	20.5	-0.2	34.2	-0.8
T_9_	21.1	36.1	20.9	0.2	35.3	0.8
T_10_	21.8	31.9	22.0	-0.2	32.7	-0.8
T_11_	19.9	31.6	22.4	-2.5	31.6	0
T_12_	25.5	33.0	25.4	0.1	33.0	0
T_13_	25.1	33.9	25.1	0	33.9	0

Y_1_- actual white pulped dragon fruit yields, Y_2_- actual purple-red pulped dragon fruit yields, Y_1p_-predicted white pulped dragon fruit yields and Y_2p_-predicted purple-red pulped dragon fruit yields obtained from the second-order polynomial models developed in the study for both the types (Equations 2, 3).

### 3.5 Adequacy of the models

#### 3.5.1 Residual analysis

The non-significance of the F-values for lack of fit (p < 0.05) strongly suggests competency of the model. To confirm this, residual analysis was performed, affirming the model’s capability to sufficiently approximate the values (**Table 5**). Merely relying on R² and modified R² is insufficient to warrant the reproducibility of Response Surface Methodology (RSM) outcomes. Hence, a run test was executed, yielding Z-values below 1.96 (Kenney and Keeping, 1962) for both Y_1_ and Y_2_. This indicates that residual values in the model are randomly distributed. Moreover, the Shapiro-Wilk test revealed that both Y_1_ and Y_2_ response variables approximate unity, indicating normal residual distribution. These findings collectively bolster the confidence in the RSM models developed within this study.

## 4 Conclusion

This experiment demonstrates the successful implementation of RSM to foretell the dragon fruit yield response under variable nutrient doses using a Box-Behnken Design. A second-order polynomial models are established for the response variable (Dragon fruit yield), and the obtained equation can be utilized to determine the optimum doses of N:P:K for desirable dragon fruit yield. The findings reveal that the optimum doses of N: P_2_O_5_: K_2_O for achieving higher yield in white (25.5t ha^-1^) and purple-red (35.6t ha^-1^) dragon fruits are 400:300:650 and 700:400:350 g/pillar/year, respectively. Moreover, the non-significant F value for the lack of fit (P <0.05) indicates the competence of the model. Graphical methods, in conjunction with RSM, are instrumental in establishing optimal doses that can be experimentally verified and reproducible. In nutshell, this study provides invaluable assistance to farmers in effective management nutrients for increasing dragon fruit yields. As a result, cultivation area of dragon fruits can expand and ultimately boosts the production and farm income.

## Data Availability

The original contributions presented in the study are included in the article/supplementary material. Further inquiries can be directed to the corresponding authors.
